# Selenium status in adults and children in Lusaka, Zambia

**DOI:** 10.1016/j.heliyon.2022.e09782

**Published:** 2022-06-24

**Authors:** Kanekwa Zyambo, Phoebe Hodges, Kanta Chandwe, Caroline Cleopatra Chisenga, Sebean Mayimbo, Beatrice Amadi, Paul Kelly, Violet Kayamba

**Affiliations:** a Tropical Gastroenterology & Nutrition Group, University of Zambia School of Medicine, Lusaka, Zambia; b Queen Mary University of London, London, UK; c University of Zambia School of Nursing Sciences, Lusaka, Zambia

**Keywords:** Selenium, Selenium deficiency, Zambia

## Abstract

**Background:**

Selenium (Se) is a trace element found in many foodstuffs and critical for antioxidant and immune functions. Widespread Se deficiency has been noted in populations of some sub-Saharan African countries including Ethiopia and Malawi. As a first step towards developing a fuller understanding of problems with the availability of Se in the diet in Lusaka province, Zambia, we measured plasma Se in adults and children in this geographic area.

**Methods:**

Total plasma Se was measured using inductively coupled plasma optical emission spectrometry (ICP-OES) in several groups of adults recruited to various pre-existing studies, including those of high and low socioeconomic status (SES) and pregnant women, and children with a range of nutritional states (healthy, stunted or wasted).

**Results:**

A total of 660 plasma samples from 391 adults and 269 children were included. Adults had a median plasma Se concentration of 0.27 μmol/l (IQR 0.14–0.43). Concentrations consistent with deficiency (<0.63 μmol/l) were found in 83% of adults. Low SES was associated with low plasma Se among adults, [OR 0.1; 95% CI 0.1–0.3, p < 0.0001]. Among the children, 24% had plasma Se less than 0.41 μmol/l. There was a statistically significant positive correlation between plasma Se and age among children, Spearman's rho 0.47, p < 0.0001.

**Conclusions:**

These data suggest that Se deficiency is widespread in Lusaka province and could in part be related to socio-economic status. Supplementation or agronomic biofortification may therefore be needed.

## 1 Introduction

Selenium (Se) is a micronutrient required for the function of an important group of Se-dependent enzymes and proteins, including glutathione peroxidases and iodothyronine deiodinases [1]. Selenocysteine, a modified amino acid, is the most abundant form of Se in the body, central to the function of 25 selenoproteins [2]. Absorption of Se by the body for use in these selenoproteins is dependent on the form of Se ingested, with absorption from organic Se considerably more efficient than inorganic Se [3].

Se deficiency has been observed in several countries throughout the world. Se status in human populations is dependent on local soils and agriculture (at least in populations where a large proportion of the diet is grown and sourced locally as in Zambia) as people that live in areas with soils that are Se deficient tend to be deficient [4, 5]. The consequences of short- and long-term Se deficiency in humans are still uncertain, but several clinical syndromes have been described. Keshan disease, a cardiomyopathy, and Kashin-Beck disease, an osteochondropathy, were both described in China [6] as conditions directly associated with Se deficiency (although neither has been described in Zambia to our knowledge). Se deficiency has also been associated with other conditions such as increased viral virulence, decreased immune function and thyroid autoimmune disease [7]. The role of Se in immune function may be particularly relevant for populations where Human Immuno Deficiency Virus (HIV) is endemic, as studies prior to the advent of antiretroviral therapy suggested that Se deficiency was associated with a higher risk of mortality in HIV [8]. More recently, meta-analysis of trials comparing Se supplementation with placebo in HIV-infected individuals found that supplementation delayed CD4 decline, although no effect of Se supplementation on viral load was found [9]. In addition to its reported associations with outcomes in HIV, Se status has also been linked to prognosis in COVID-19 infection, with data from a European cross-sectional analysis showing that Se status was significantly higher in COVID-19 survivors as compared to non-survivors [10]. A study of the effect of Se supplementation on immune response to oral live attenuated polio vaccine in individuals with marginally low Se levels in the UK found that supplementation augmented cellular immune responses, significantly increasing percentage of CD4+ T cells and leading to an earlier peak T cell proliferative response [11]. This finding may be particularly important for populations of low-income countries where environmental enteropathy is widespread. Environmental enteropathy is a structural and functional abnormality of the small bowel thought to represent an adaptation to epithelial damage caused by recurrent exposure to enteropathogens, which is known to reduce the efficacy of oral vaccines including polio [12].

In addition to its role in immune function, Se also has a neuroprotective function through the role of selenoprotein P in delivery of Se to the brain, with risk of Alzheimer's and dementia being linked to Se status [2]. High Se exposure has also been found to reduce the risk of certain cancers including breast, lung, oesophageal, gastric, and prostate cancers [13].

Data on Se status from populations in sub-Saharan Africa are still scanty. In Africa, soil Se concentrations are extremely varied, with some countries such as Ethiopia and Malawi showing large variations between regions [14]. In a study conducted in Ethiopia on cognitive performance, Se deficiency was observed in children who scored lower on cognitive tests [15]. Studies in South Africa [16] and Malawi [17] indicated that Se deficiency was widespread in this region. A study in HIV-infected adults in Botswana also suggested that deficiency was common [18]. In Zambia, previous studies have reported low blood Se levels in children [19].

In order to contribute to a fuller understanding of Se deficiency in Lusaka province, we measured plasma Se concentrations in several groups of adults and children.

## 2 Methods

This was a cross-sectional study using samples collected during the conduct of previously reported and unreported studies in adults and children in Lusaka province, Zambia (Table 1). Plasma samples therefore fell into several groups: adults of low socioeconomic status (SES), adults of relatively high SES, endoscopic controls from a study of gastric cancer, pregnant women, children with severe acute malnutrition (SAM), children with stunting (length for age z score < -2), and children without stunting. All children from these studies came from high-density residential areas of Lusaka with populations known to be at risk of malnutrition and environmental enteropathy based on previous studies, hence by this definition were of lower SES. These studies were all separately approved by the University of Zambia Biomedical Research Ethics Committee (UNZABREC), with reference numbers shown under each group. Informed consent was obtained from all the participants/legal guardians (in case of participants below age 18) involved in the study.

**Table 1:** Summary of participants and studies from which they were drawn.

Participant group	Recruitment dates	Recruitment approach	Inclusion/exclusion criteria	Participants included in current study	Reference
Adults of low SES	Jun 2013–Apr 2014	Healthy volunteers were recruited from Misisi compound (an unplanned high-density settlement in Lusaka) via a 3-stage consent process	Inclusion: adults giving informed consent Exclusion: concurrent illness, pregnancy, use of antibiotics or NSAIDs within 1 month, recent helminth infection detected in a single stool sample	All participants from this study were included in the current study	Kelly P et al, 2016, ‘Endomicroscopic and transcriptomic analysis of impaired barrier function and malabsorption in environmental enteropathy’, PLoS Negl Trop Dis, 10(4):e0004600
Adults of high SES	Feb–Mar 2015	Participants were identified among staff of University Teaching Hospital, Lusaka	Inclusion: 18–65 yrs old; in good health, with normal full blood count and normal nutritional status Exclusion: seropositivity for HIV, Hepatitis C, HBsAg, or syphilis; participating in another study.	All participants from this study were included in the current study	Chisenga C, Kelly P, 2016, ‘T cell subset profile in healthy Zambian adults at the University Teaching Hospital’, Pan Afr Med J, 23:103
Endoscopic controls from gastric cancer study	Jul 2016–Apr 2018	Patients presenting to the endoscopy unit at University Teaching Hospital, Lusaka for oesophagogastro-duodenoscopy were invited for enrolment	Inclusion: patients who gave written informed consent were included Exclusion: individuals with prior history of gastric or oesophageal cancer	Controls from this study (patients without suspicious lesions at endoscopy and without any detectable gastric premalignant lesions seen on histology) were included in the current study	Kayamba V et al, 2020, ‘Biomass smoke exposure is associated with gastric cancer and probably mediated via oxidative stress and DNA damage: a case-control study’, JCO Glob Oncol, 6:532–41
Pregnant women	Dec 2017–Jun 2018	Women were invited when attending for antenatal care at Chilenje Hospital, Lusaka	Inclusion: women in second or third trimester who consented to participate. Exclusion: blood transfusion in previous 3 months; pre-existing haematological condition; certain medications (calcium and zinc supplements, cisplatin)	All participants from this study were included in the current study	Mayimbo S et al, 2018, ‘Micronutrient status as predictors of low birth weight and pre-term delivery in women attending antenatal care in Chillenje, Lusaka, Zambia. A proposal for research’, IOSR-JNHS, 7(2): 22-27
Children with SAM	May 2014–Feb 2015	Children hospitalised with SAM and persistent diarrhoea (3 or more loose stools per day for 14 days or more) in the University Teaching Hospital, Lusaka, were enrolled in the study, as well as groups of healthy adults and children from the community	Inclusion: signed informed caregiver consent Exclusion: exclusion criteria for the healthy adults and children in this study were recent episode of diarrhoea within 1 month, NSAID or antibiotic use within 1 month	All children with SAM from the pre-existing study were included in the current study but not the community children. The adults described in this study represent the group ‘Adults of low SES’ in the current study	Amadi B et al, 2017, ‘Impaired barrier function and autoantibody generation in malnutrition enteropathy in Zambia’, EBioMedicine, 22:191–99
Children with stunting	Aug 2016–Jun 2019	Children from Misisi compound were screened using anthropometric measures as part of the BEECH (Biomarkers of enteropathy in children) study. Undernourished children were given nutritional rehabilitation and well-nourished children were recruited as controls	Inclusion: inclusion criteria for the group included in the current study were caregiver consent and length-/height-for-age ​z ​score of < –2 SD Exclusion: nil	Controls from the BEECH study are included in the current study	Amadi B et al, 2021, ‘Adaptation of the small intestine to microbial enteropathogens in Zambian children with stunting’, Nat Microbiol, 6:445–54
Children without stunting	May 2014–Feb 2015, and Aug 2016–Jun 2019	These children were recruited from the BEECH study as described above and also during the course of an earlier community malnutrition screening programme conducted in Misisi and Kuku compounds in Lusaka	Inclusion: inclusion criteria for controls (the group included in the current study) were anthropometric evidence of good growth and informed caregiver consent Exclusion: recent episode of diarrhoea within 1 month; NSAID use or antibiotic use within 1 month	The participants in the current study are the healthy controls from these two studies (children with no evidence of stunting)	Amadi B et al, 2017, ‘Impaired barrier function and autoantibody generation in malnutrition enteropathy in Zambia’, EBioMedicine, 22:191–99, Amadi B et al, 2021, ‘Adaptation of the small intestine to microbial enteropathogens in Zambian children with stunting’, Nat Microbiol, 6:445–54

## 3 Participants and samples

### 3.1 Adults of low socioeconomic status

Samples from 61 adults from a previously published community study of environmental enteropathy [20] were included. Ethics approval was obtained from UNZABREC, reference number 006-01-13. Misisi compound is a high-density residential area of very low socio-economic status, with poor sanitation, very limited dietary diversity, and inadequate access to food refrigeration facilities. Volunteers with concurrent illness, pregnancy or use of antibiotics and nonsteroidal anti-inflammatory drugs within one month, helminth infection detected in stool sample, or currently involved in other studies were excluded.

### 3.2 Adults of relatively high socioeconomic status

Samples from a previously published study of 49 adults who were professional and technical full-time employees in the University Teaching Hospital were included. These individuals were in formal employment, with job titles such as accountant, doctor, or laboratory scientist, and their occupational status was used to define this as a higher socio-economic group than the others [21]. Ethics approval was obtained from UNZABREC, reference number 009-01-11. Participants were physically active, with normal full blood count parameters and in good health. Exclusion criteria were HIV seropositive, HBsAg seropositive, hepatitis C virus seropositive, syphilis seropositive, participating in another conflicting study, pregnancy or having any chronic disease.

### 3.3 Endoscopic controls from gastric cancer study

This group included 85 controls drawn from a previously published study [22] of gastric cancer (UNZABREC, reference number 005-03-16), where controls were individuals who attended for diagnostic endoscopy at the University Teaching Hospital, Lusaka. These individuals had no endoscopic or histological evidence of malignancy. Although these adults were not from the same low socioeconomic group as the participants from Misisi compound, since they were attending a government hospital it is likely that they were not from the highest socioeconomic group in Zambia as this group tends to seek healthcare from private providers.

### 3.4 Pregnant women

We analysed samples from 196 pregnant women participating in a study of risk factors for low birth weight among antenatal mothers [23]. These were women from varying socio-economic backgrounds attending the antenatal clinic at Chilenje Hospital in Lusaka. Women who had haematological conditions such as sickle cell anaemia, were on zinc supplements or who had a history of blood transfusion in the three months before the study were excluded. Ethics approval was obtained from UNZABREC reference number 007-11-17.

### 3.5 Children with SAM

We included data from 19 children with SAM and persistent diarrhea [24]. These children all received standard nutritional rehabilitation during their stay in hospital, which included a micronutrient supplement (selenium 47 μg/100 ml, CMV, Nutriset, Malauney, France) although data pertaining to exact doses received by each child during the course of their hospitalisation is not available. Ethics approval was obtained from UNZABREC, reference number 006-01-13.

### 3.6 Children with stunting

We analysed samples from 164 children with stunting (length for age z score < -2), enrolled in a longitudinal study on biomarkers of environmental enteropathy in Misisi compound [25]. These children had repeated measurements for weight, length, and mid-upper arm circumference, were offered nutritional support, including corn-soy blend, a daily egg, and a micronutrient sprinkle containing selenium 17 mg/day as sodium selenite (Nutromix, Hexagon Nutrition, Chennai, India), and underwent investigation for non-response. Ethics approval was obtained from UNZABREC, reference number 006-02-16.

### 3.7 Children without stunting

Eighty-six children from community studies on environmental enteropathy who were not stunted (defined by z scores of > -2) were also included [24, 25]. These children were recruited from two separate studies (UNZABREC, reference numbers 006-01-13 and 006-02-16) and were all from the same high-density residential areas in Lusaka as the children with stunting, where environmental enteropathy is known to be widespread. They were physically active, in good health, tested negative for helminth infections and had no diarrhoea during the two weeks prior to the date of recruitment. These children were not given supplements.

### 3.8 Measurement of plasma Se

Total plasma Se was measured using inductively coupled plasma optical emission spectrometry (ICP-OES) within the Tropical Gastroenterology and Nutrition Group (TROPGAN) labs at University of Zambia School of Medicine, Lusaka. In all cases, blood samples were collected in trace element free tubes (TEKLAB, Sacriston, Co Durham, UK). Blood was centrifuged at 856g for 15 minutes at 4°C to extract plasma, then rapidly stored in a −80 °C freezer. Plasma Se levels were measured using the Optima DV 7000 (PerkinElmer, Midrand, South Africa). Diluent containing 100ml 2% Nitric acid, 1ml 3M HCl and 99ml 1% Triton was used to digest the sample. Five working standard solutions ranging from 39.4-196 μg/L and a blank (PerkinElmer Pure Instrument Calibration Standard 4-N930021) was used to create a standard curve. Seronorm L-1 (SERO AS LOT 0903106; range 93–121 μg/L) 1:9 v/v with diluent and a pooled plasma sample were used as quality control (QC) which was rerun after 10 samples. Recovery rate from standard reference material was 102–119 μg/L. Plasma samples were diluted (1:9 v/v) with diluent. Instrument working conditions were set at: wavelength 196.026nm; plasma gas flow 1.5 L/min; auxiliary gas flow 0.2 L/min; nebulizer gas flow 0.6 L/min; RF Power 1450 W; plasma view: axial; read delay: 90 s; read parameters: 2.0 min, 5.0 max; peristaltic pump flow rate 1.8 ml/min, and three replicates were analysed per sample. The software was able to determine and/or exclude any outliers.

## 4 Data analysis

Results are shown as median and interquartile range (IQR) as Se concentrations were not normally distributed, tested using the Shapiro Wilk test. Mann Whitney U and Kruskal Wallis H tests were used for comparison of non-parametric variables between groups. Spearman rank-order correlation coefficient was used for correlation between age and selenium concentration. In all cases, p-values less than 0.05 were considered statistically significant. Data were analysed in SPSS version 26.

## 5 Results

Plasma Se was measured in 660 samples: 391 adults with a median (IQR) age of 30 (IQR 24–40) years (Table 2), and 269 children, with a median age of 18 (IQR 12–22) months (Table 3).

**Table 2:** Characteristics of adults included in the study.

	High SES (n = 49)	Low SES (n = 61)	Endoscopic controls (n = 85)	Pregnant women (n = 196)
Age	29 (25-37)	27 (23–38)	50 (41–60)	26 (23-32)
Female (%)	36.7	70.7	54.1	100
BMI (kg/m^2^)	23.5 (21.6–26.4)	22.5 (20.7–25.3)	24.3 (21.0–27.1)	-
Selenium concentration (μmol/L)	1.38 (0.87–1.53)	0.28 (0.22–0.41)	0.28 (0.14–0.66)	0.16 (0.09–0.37)
Selenium concentration (μg/L)	108.7 (68.6–120.7)	21.8 (17.2–32.0)	22.3 (11.1–51.6)	12.9 (7.4–29.2)

Results are shown as median (IQR).

**Table 3:** Characteristics of children included in the study. Children with severe acute malnutrition and stunting were receiving Se supplementation.

	Severe acute malnutrition (n = 19)	Stunting (n = 164)	No stunting (n = 86)
Age (months)	18 (12-21)	19 (15-23)	8.5 (4-22)
Length for age z score	-3.10 (-3.94–2.32)	-3.24 (-3.78–2.65)	-0.55 (-1.19–0.11)
Weight for age z score	-3.60 (-5.22–3.10)	-2.18 (-2.70–1.64)	-0.78 (-1.23–0.32)
Weight for length z score	-2.94 (-4.51–2.56)	-0.63 (-1.29–0.00)	0.21 (-0.40–0.95)
Selenium concentration (μmol/L)	0.91 (0.60–1.13)	0.57 (0.53–0.82)	0.41 (0.26–0.79)
Selenium concentration (μg/L)	71.5 (48.3–88.6)	45.0 (41.6–64.3)	32.4 (20.8–62.4)

Results are shown as median (IQR).

### 5.1 Adults

**Figure 1:**
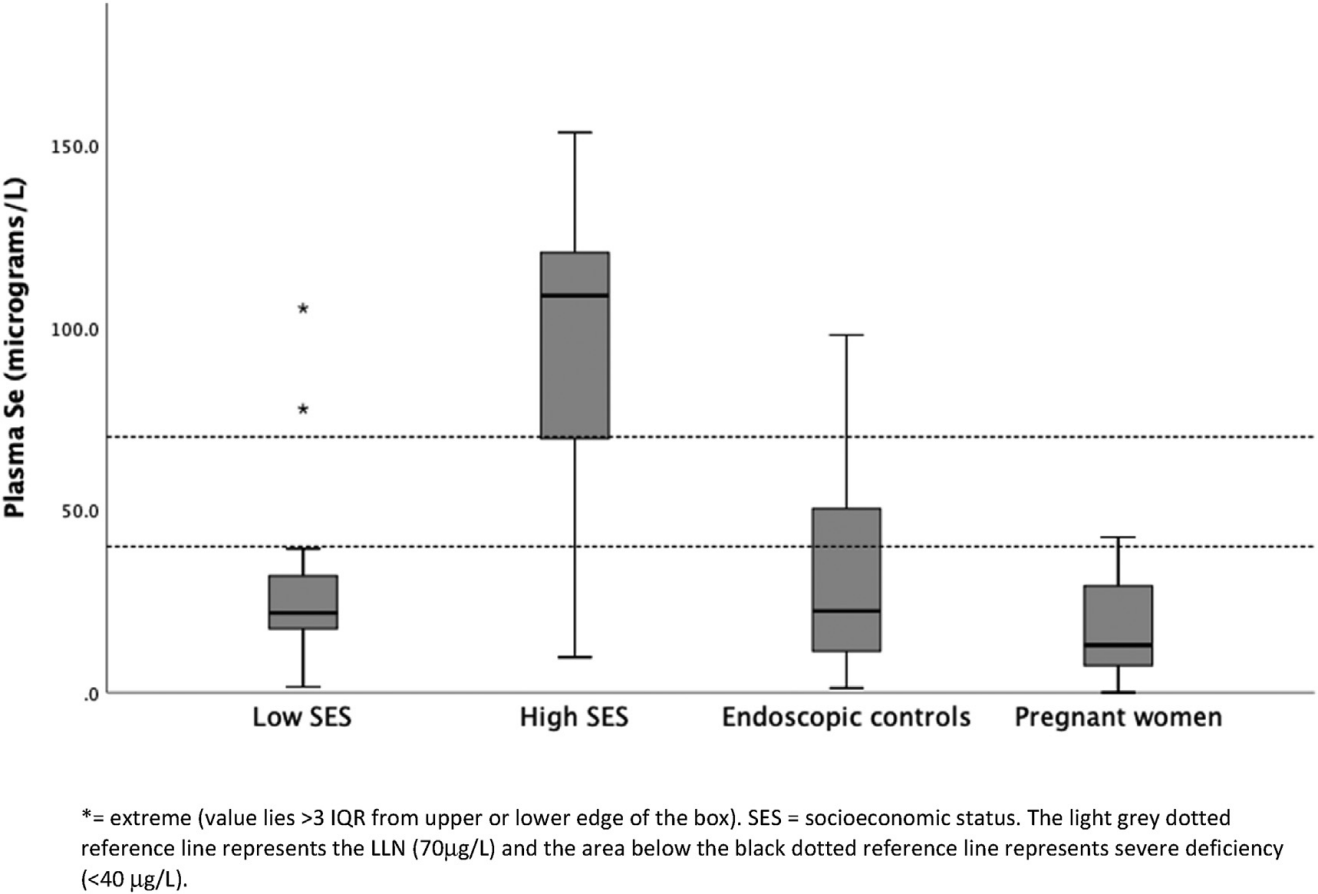
Plasma selenium concentrations in adults. ∗ = extreme (value lies >3 IQR from upper or lower edge of the box). SES = socioeconomic status. The light grey upper dotted reference line represents the LLN (70 μg/L) and the area below the lower dotted reference line represents severe deficiency (<40 μg/L).

Characteristics of adults are shown in Table 2. The median plasma Se among adults was 0.27 μmol/l (IQR 0.14–0.43) or 21.2 μg/L. Adults of high and low SES were of similar age and BMI but plasma Se concentrations were significantly higher in adults of high SES (p < 0.001) (Table 2 and Figure 1). Using 0.63 μmol/l as the lower limit of normal (LLN) [26] 83% of adults had low plasma Se, or using 70 μg/L as the LLN, 88% of adults had low plasma Se. A study conducted in Germany found that a serum Se of <45.7 μg/L represented the 2.5th percentile of the reference population [10]. Assuming that a plasma Se level of <40 μg/L represents severe deficiency, 82% of adults in our study were severely deficient. However, only 64% of adults of high SES had plasma Se concentrations below the LLN compared to 92% of adults of low SES, [OR 0.1; 95% CI 0.1–0.3, p < 0.0001] (using the molar definition of LLN). All of the pregnant women had Se concentrations below the LLN regardless of which definition was used, and 99% were severely deficient with a plasma Se concentration of <40 μg/L.

### 5.2 Children

**Figure 2:**
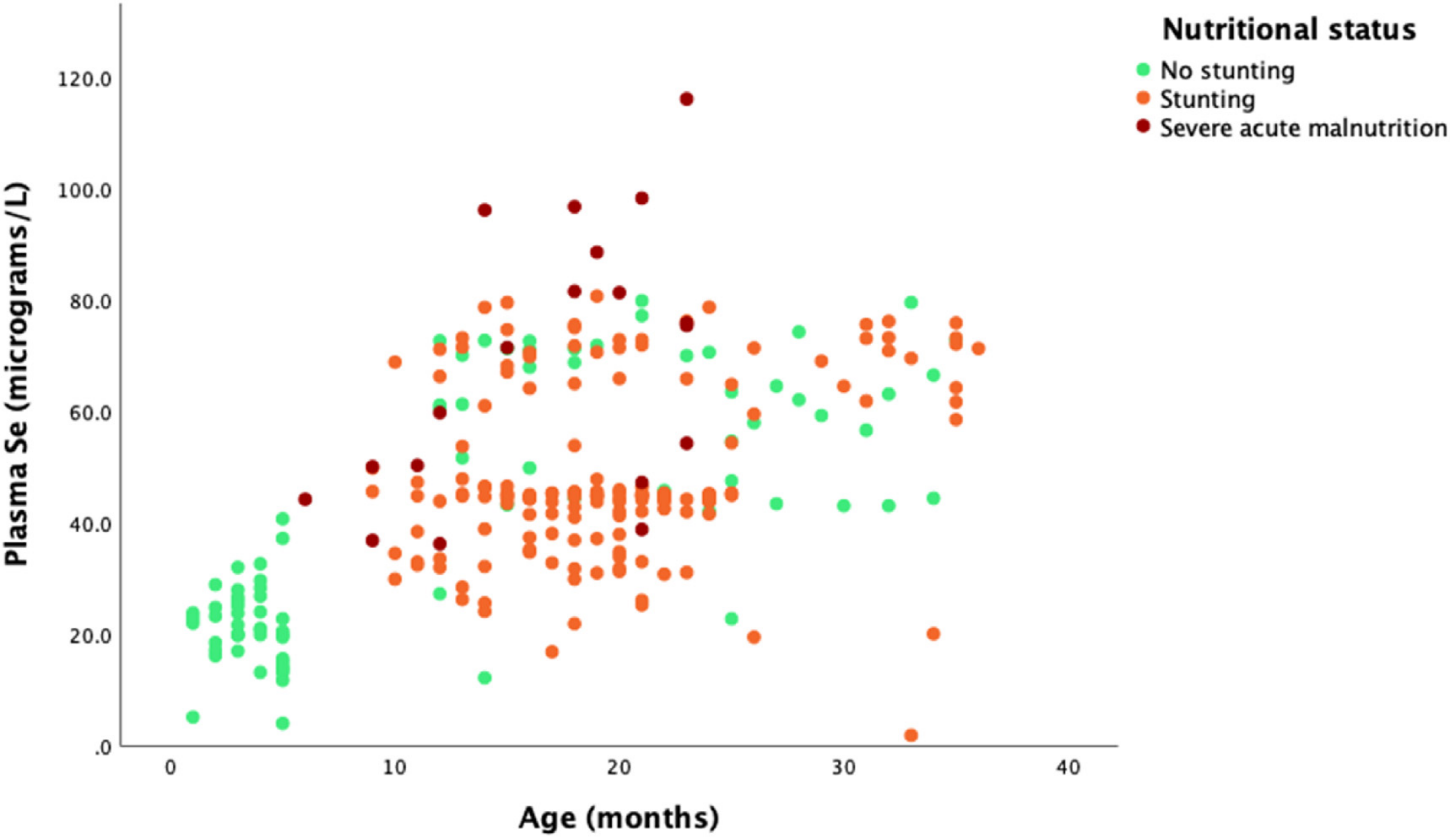
Plasma selenium concentration in children at different ages. Children with stunting and severe acute malnutrition were receiving Se supplementation.

**Figure 3:**
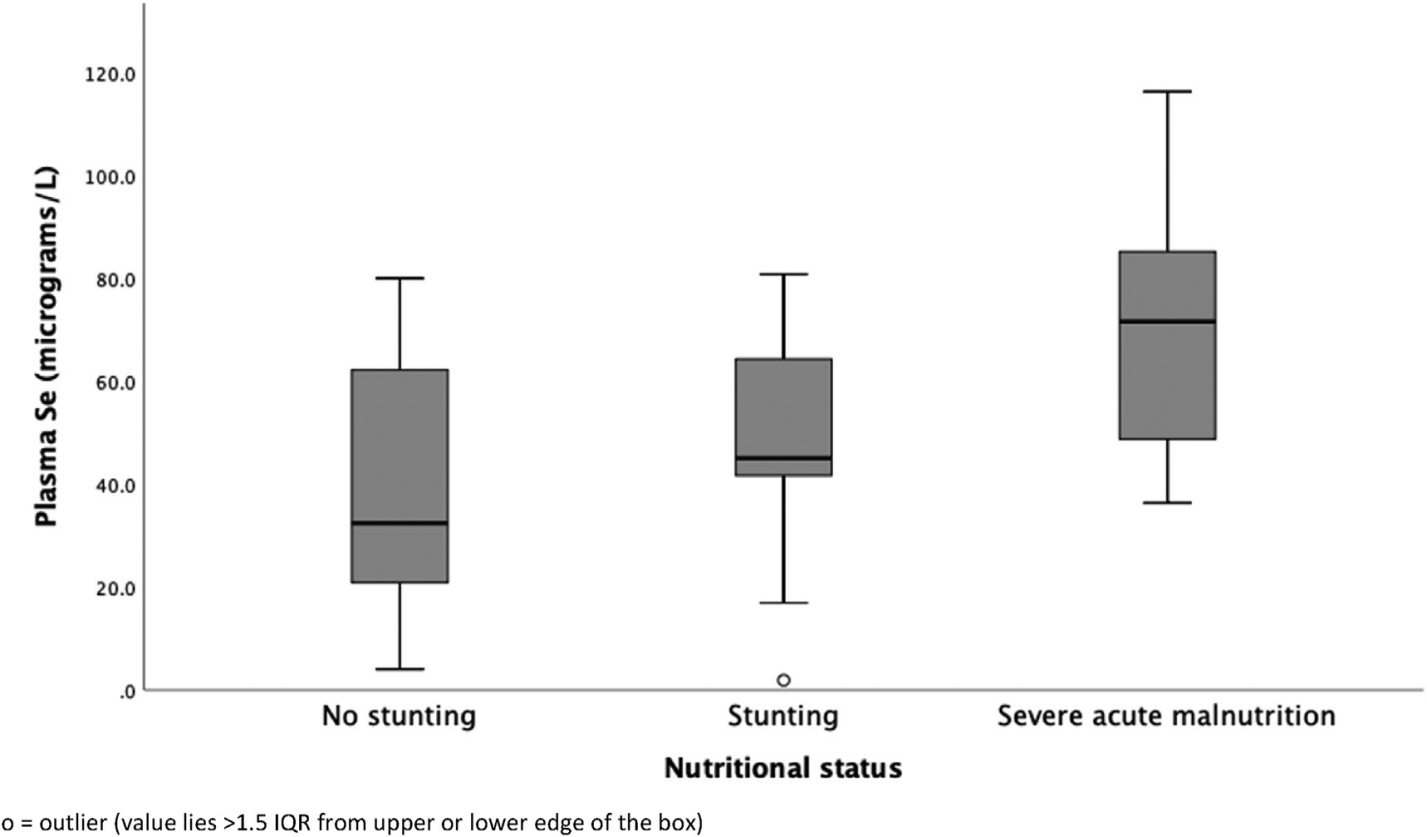
Plasma selenium concentrations in children grouped by nutritional status. Children with stunting and severe acute malnutrition were receiving Se supplementation.

Plasma Se concentrations were measured in 269 children as shown in Figure 2. Using 0.41 μmol/l as the LLN for children [26], 24% of them had low plasma Se. However, 132 (49%) of these children were taking nutrient supplementation which contained Se. The percentage of low Se among supplemented children was 11% while it was 36% among those not being supplemented, a difference which was statistically significant, [OR 0.3; 95% CI 0.1–0.6, p < 0.0001]. There was a significant correlation between Se and age, with plasma Se concentration increasing with age, Spearman's rho 0.47, p < 0.0001 (Figure 2). The median Se levels were 0.41 μmol/l (0.27–0.79) among unstunted children, 0.57 μmol/l (0.53–0.82) among stunted children and 0.91 μmol/l (0.60–1.13) among severely malnourished children. The difference across these groups was statistically significant (p < 0.001; Figure 3).

## 6 Discussion

Se deficiency is widespread in the groups included in this analysis and socio-economic status appears to have an effect on Se status although given the design of the current study it is not possible to draw definite conclusions about this. Adults in formal employment in high-status jobs in the main teaching hospital had higher plasma Se concentrations, presumably reflecting access to more diverse food sources allowing increased consumption of high Se-containing foods such as fish and beef liver, which would not be readily available to low SES groups. This finding was consistent with data from the Malawi Micronutrient Survey, where Se deficiency was found to be associated with household wealth [17].

Pregnant women had the lowest plasma selenium concentrations of any group, and this finding is of particular public health concern as low selenium levels have been associated with adverse pregnancy outcomes [27]. In the Malawi Micronutrient Survey, the median plasma Se level in women of reproductive age was 78.4 ng mL-1 (equivalent to 78.4 μg/L), whereas the highest plasma Se level recorded in a pregnant woman in this study was 42.5 μg/L. Studies in Europe have found great variation of Se status in pregnancy, with one study from Poland finding that plasma Se concentration decreased from 48.3 ± 10.6 μg/l in the first trimester to 38.4 ± 11.8 μg/l at delivery [28], whereas a study conducted in women in the first trimester of pregnancy in Spain found a mean serum Se level of 79.7 μg/l [29]. In a study of HIV-positive pregnant women in Nigeria, 20.4 % were found to have selenium deficiency, where deficiency was defined as serum Se < 0.89 μmol/l (compared to 100% of pregnant women in our study) and these women had an eight-fold higher risk of preterm delivery [30]. A randomised controlled-trial of prenatal Se supplementation from the same group found a beneficial effect of this intervention as women who received supplements had a significantly lower risk of preterm delivery [31].

It is not surprising that plasma Se concentrations appear to be higher in children with SAM and in children who were stunted than in children without stunting as the former two groups were receiving Se supplementation. It should also be noted that, although both the SAM group and the group with stunting were receiving Se supplementation, the SAM group had higher plasma concentrations of selenium than the stunting group. This may be due to differences in administration and Se content of the supplements, with children with SAM receiving inpatient nutritional rehabilitation, whereas children with stunting were given a micronutrient sprinkle in the community where it is possible that adherence was low.

Variations in Se levels in healthy children with no growth faltering were observed, but the clinical implications of low levels in these children are not clear. It was clear that plasma Se concentration increased with age but one potential confounding factor in this study was that the children in the group with no growth faltering were younger than in the other two groups (median 8.5 months compared to a median of 18 months in the SAM group and 19 months in the group with stunting, although this difference did not reach significance). A previous study of age-related serum Se concentrations in a cohort of 1010 apparently healthy children in Germany found a statistically significant age dependency, with levels decreasing from the age of <1 month to 4 months, then increasing to 0.62 μmol/L between the ages of 4–12 months before plateauing at 0.9 μmol/L up to the age of 5 years [32]. Based on this data, it seems that children with SAM (median age 18 months, median Se 0.91 μmol/L) had comparatively normal Se levels in contrast with children in the other two groups.

The widespread Se deficiency in the groups studied would be expected to result in attributable morbidity, particularly with regards to pregnancy outcomes, and possible mortality. In Zambia, cardiomyopathy accounts for 75% morbidity and 30% mortality of all cases seen at the Adult Medicine Hospital in Lusaka (University Teaching Hospital Adult medicine records). Se deficiency is known to be involved in Keshan disease, a severe form of cardiomyopathy endemic to China [33], and a study in Nigeria showed that Se levels of less than 70 μg/L was a risk factor for development of peripartum cardiomyopathy [34]. Given that 99% of pregnant women in our study had plasma Se < 40 μg/L, this is a point of particular concern. The link between Se deficiency and cardiomyopathy is likely to be related to the role of Se in regulating cardiomyocyte apoptosis, although the negative impact of Se deficiency on redox regulation, thyroid hormone metabolism, and calcium flux due to selenoprotein involvement in these pathways may also contribute [35]. Whether the high incidence of cardiomyopathy in Zambia can be corrected by Se supplementation requires further study.

One limitation of this study with regards to the adult groups is that participants were drawn from different studies, hence we were not able to define socioeconomic status for all participants. However it is likely that the endoscopic controls from the gastric cancer study lie somewhere between the adults in the low SES and high SES groups. Relative strengths of this study include the large numbers of participants involved and the broad spectrum in terms of socioeconomic status of participants, as well as the plausibility of results given what is already known about selenium bioavailability and human selenium deficiency in relation to socioeconomic status in other sub-Saharan African countries [17, 36].

Our data suggest that Se deficiency is widespread in Lusaka province and is related at least in part to socio-economic status. These findings are of significant concern given the possible implications of Se deficiency for human health and growth and may imply a need for remedial approaches such as agronomic biofortification of maize as has been undertaken elsewhere in order to address population Se deficiencies [37]. However, these data are not generalisable to the whole of Zambia given that Se status has been shown in previous studies to be under geospatial control [17], and there is likely to be variation in soil Se bioavailability and hence in dietary Se throughout the country. Further investigation is warranted in order to increase understanding of these factors and how they may contribute to Se levels in the wider Zambian population.

## Declarations

### Author contribution statement

Kanekwa Zyambo: Conceived and designed the experiments; Performed the experiments; Analyzed and interpreted the data; Wrote the paper.

Phoebe Hodges: Analyzed and interpreted the data; Wrote the paper. Kanta Chandwe, Caroline Cleopatra Chisenga, Sebean Mayimbo, Beatrice Amadi: Contributed reagents, materials, analysis tools or data; Wrote the paper.

Paul Kelly: Conceived and designed the experiments; Analyzed and interpreted the data; Contributed reagents, materials, analysis tools or data; Wrote the paper.

Violet Kayamba: Analyzed and interpreted the data; Contributed reagents, materials, analysis tools or data; Wrote the paper.

### Funding statement

Paul Kelly was supported by 10.13039/100000865Bill and Melinda Gates Foundation [OPP1066118]. Violet Kayamba was supported by 10.13039/100000061Fogarty International Center [D43 TW009744]. Caroline Cleopatra Chisenga was supported by the 10.13039/501100001713European and Developing Countries Clinical Trials Partnership (IP.2009.33011.004).

### Data availability statement

Data included in article/supp. material/referenced in article.

### Declaration of interests statement

The authors declare no conflict of interest.

### Additional information

No additional information is available for this paper.
